# Integrative Pan-Cancer Analysis of KIF15 Reveals Its Diagnosis and Prognosis Value in Nasopharyngeal Carcinoma

**DOI:** 10.3389/fonc.2022.772816

**Published:** 2022-03-11

**Authors:** Jinglin Mi, Shanshan Ma, Wei Chen, Min Kang, Meng Xu, Chang Liu, Bo Li, Fang Wu, Fengju Liu, Yong Zhang, Rensheng Wang, Li Jiang

**Affiliations:** ^1^ Department of Radiation Oncology, The First Affiliated Hospital of Guangxi Medical University, Nanning, China; ^2^ Department of Oncology, Yunfu People’s Hospital, Yunfu, China; ^3^ Key Laboratory of High-Incidence-Tumor Prevention & Treatment (Guangxi Medical University), Ministry of Education, Nanning, China; ^4^ Department of Pathology, The First Affiliated Hospital of Guangxi Medical University, Nanning, China

**Keywords:** KIF15, pan-cancer analysis, nasopharyngeal carcinoma, diagnosis, prognosis

## Abstract

**Background:**

KIF15 plays a vital role in many biological processes and has been reported to influence the occurrence and development of certain human cancers. However, there are few systematic evaluations on the role of KIF15 in human cancers, and the role of KIF15 in the diagnosis and prognosis of nasopharyngeal carcinoma (NPC) also remains unexplored. Therefore, this study aimed to conduct a pan-cancer analysis of KIF15 and evaluate its diagnostic and prognostic potential in NPC.

**Methods:**

The expression pattern, prognostic value, molecular function, tumor mutation burden, microsatellite instability, and immune cell infiltration of KIF15 were examined based on public databases. Next, the diagnostic value of KIF15 in NPC was analyzed using the Gene Expression Omnibus (GEO) database and immunohistochemistry (IHC). Kaplan–Meier curves, Cox regression analyses, and nomograms were used to evaluate the effects of KIF15 expression on NPC prognosis. Finally, the effect of KIF15 on NPC was explored by *in vitro* experiments.

**Results:**

The expression of KIF15 was significantly upregulated in 20 out of 33 cancer types compared to adjacent normal tissue. Kyoto Encyclopedia of Genes and Genomes enrichment (KEGG) analysis showed that KIF15 could participate in several cancer-related pathways. The increased expression level of KIF15 was correlated with worse clinical outcomes in many types of human cancers. Additionally, KIF15 expression was related to cancer infiltration of immune cells, tumor mutation burden, and microsatellite instability. In the analysis of NPC, KIF15 was significantly upregulated based on the GEO database and immunohistochemistry. A high expression of KIF15 was negatively associated with the prognosis of patients with NPC. A nomogram model integrating clinical characteristics and KIF15 expression was established, and it showed good predictive ability with an area under the curve value of 0.73. KIF15 knockdown significantly inhibited NPC cell proliferation and migration.

**Conclusions:**

Our findings revealed the important and functional role of KIF15 as an oncogene in pan-cancer. Moreover, high expression of KIF15 was found in NPC tissues, and was correlated with poor prognosis in NPC. KIF15 may serve as a potential therapeutic target in NPC treatment.

## Introduction

Cancer has become the leading cause of morbidity and mortality in low- and high-income countries around the world (1). Due to population aging and growth, the global number of patients with cancer is predicted to increase (2). Despite great advances in diagnostic and therapeutic methods of treating cancer in recent years, the survival outcome and quality of life of patients remains unsatisfactory (3). Among all types of cancers, nasopharyngeal carcinoma (NPC) is endemic to southeast Asia, north Africa, and southern China. According to GLOBOCAN estimates, the age-standardized rate of NPC is 4–25 cases per 100,000 individuals in these regions (4, 5). The tumorigenesis and progression of NPC are closely related to genetic factors, environmental effects, and Epstein–Barr virus (EBV) infection (6). Advanced NPC had worse clinical outcomes due to delayed diagnosis and distant metastasis are the critical factors for treatment failure (7). Consequently, it is necessary to identify novel biomarkers and investigate the molecular mechanisms for improving early diagnosis and prognosis of NPC.

The kinesin superfamily (KIF) is an important microtubule-dependent motor protein that participates in the transport of various cargos including vesicles, membranous organelles, and mRNAs (8). To date, more than 45 KIF members have been found in mammalian cells and they are classified into 14 families base on their structural features (9). KIF15, a member of kinesin-12 family, is a plus end-directed motor with an N-terminal motor domain that plays a key role in bipolar spindle assembly (10). During cell division, dysregulation of KIF15 can result in aberrant cell proliferation, tumorigenesis, and tumor aggressiveness (11). Recently, KIF15 has been proven to be over-expressed in various cancers including gastric cancer, hepatocellular carcinoma, and lung adenocarcinoma (12–14). However, the functions of KIF15 in pan-cancer are not fully understood. Moreover, there is a lack of evidence on the effects of KIF15 expression on the diagnosis and prognosis of NPC.

In the present study, we comprehensively analyzed the expression signature, prognostic value, and associated pathways of KIF15 across 33 types of human cancers using multiple databases. Subsequently, the correlations between KIF15 and tumor mutation burden (TMB), microsatellite instability (MSI), and immune infiltration degree were investigated. We further analyzed the mRNA expression of KIF15 in NPC tissues and normal tissues using the Gene Expression Omnibus (GEO) database. Immunohistochemical analysis (IHC) was utilized to verify the protein expression level of KIF15 and its diagnostic and prognostic value in NPC. RNA interference was conducted to silence the KIF15 expression to investigate its molecular function in the NPC cell lines. The results of our study could contribute to a better understanding of the effects of KIF15 on cancer (specifically NPC) occurrence, development, and prognosis.

## Materials and Methods

### Analysis of KIF15 Differential Expression in Pan-Cancer

The Cancer Genome Atlas (TCGA) is a web-based, publicly available database, which contains more than 2.5 petabytes of genomic, transcriptomic, and proteomic data of over 20,000 cancer patients across 33 different cancer types (http://cancergenome.nih.gov/). Gene expression data and clinical data of TCGA were downloaded using the University of California, Santa Cruz Xena (UCSC Xena) online tool. Wilcoxon test was used to assess the expression levels of KIF15 in various cancers based on TCGA database. The Oncomine database is a useful platform that provides a powerful series of analyses, including comparison gene expression signatures, clusters, and gene-set modules (www.oncomine.org). The Kaplan-Meier plotter is capable of evaluating the potential role of mRNA, miRNA, and proteins in 21 cancer types (http://kmplot.com/analysis/). The expression pattern of KIF15 in pan-cancer was further verified by Oncomine database and Kaplan-Meier plotter database. The clinical relationship between KIF15 expression level and patients’ cancer stage was evaluated using limma package and RColorBrewer package was used to visualized the results, we used ‘avereps’ function from limma package to condense the microarray data object so that values for within-array replicate probes are replaced with their average, for each cancer type, we compared gene expression differences of KIF15 between each of the two cancer stages using the Wilcox test. *P* values were set as statistically significant according to the following: *P<0.05; **P<0.01 and ***P<0.001.

### Analysis of KIF15 Expression and Prognosis in Pan-Cancer

To evaluate the KIF15 potential prognostic value in pan-cancer, univariate Cox regression and Kaplan-Meier (KM) method were used to analyze overall survival (OS), disease-free interval (DFI), disease-specific survival (DSS) and progression-free interval (PFI) based on TCGA database. The GEPIA web-based platform was applied to analyze the KIF15 expression level in pan-cancer (http://gepia2.cancer-pku.cn). A *P* value <0.05 was set as significantly different.

Meta-analysis was carried out using Review Manager (RevMan) version 5.3. The eligible studies were searched for on public databases, including PubMed, PrognoScan and Chinese National Knowledge Infrastructure (CNKI) up to December 31, 2021. The search strategy was as follows: (“KIF15” or “kinesin family member 15”) AND (“tumor” or “cancer” or “carcinoma” or “malignancy”) AND (“survival” or “outcome” or “prognostic”). The inclusion criteria were: (1) the expression level of KIF15 was detected in human cancer; (2) the correlation of KIF15 expression and OS or Disease-free survival (DFS) or Relapse-Free Survival (RFS) or Local relapse-free survival (LRFS) or Distant metastasis-free survival (DMFS) was evaluated; and (3) the hazard ratios (HRs) with 95% confidence intervals (CIs) could be acquired directly or estimated. The exclusion criteria were: (1) articles that were reviews, case reports, letters, meeting abstracts, or expert opinions; (2) duplicate literature; and (3) data that were insufficiently detailed or the needed descriptive or inferential statistics could not be calculated.

We evaluated the correlation between KIF15 and the survival results (OS, RFS, and DMFS) by the pooled HR and 95% CIs. A *P*<0.05 was regarded to be statistically significant. Higgins I^2^ statistics and the chi-square Q test were applied to analyze the heterogeneity of different studies. When the heterogeneity was statistically significant (*P*>0.1 or I^2^<50%), the fixed-effect model (FEM) was built; otherwise, the random-effect model (REM) was built. A funnel plot and the Egger test were used to evaluate publication bias.

### Functional Analysis of KIF15 Related Genes

The GEPIA database was utilized to identify the significantly related genes of KIF15 in human cancers. The correlation coefficient was calculated using the Pearson method and the top 100 genes most relevant to KIF15 were selected. GO and Kyoto Encyclopedia of Genes and Genomes (KEGG) enrichment analyses was performed to investigate the biological functions of these genes by Database for Annotation, Visualization, and Integrated Discovery (DAVID) (https://david.ncifcrf.gov/). Next, a protein–protein interaction (PPI) network was constructed and visualized by Cytoscape (version 8.2). The functional state of KIF15 in different cancer types was explored using CancerSEA database (http://biocc.hrbmu.edu.cn/CancerSEA/). CancerSEA is the first comprehensive database that offers a cancer single-cell functional state atlas; it contains 14 functional states of 41,900 cancer single cells across 25 cancer types. Association between KIF15 and functional state in various single-cell datasets was determined by a correlation strength >0.3 and a false discovery rate (FDR) (Benjamini & Hochberg) <0.05.

### Correlation Between KIF15 Expression and Tumor Immunity

The Tumor Immune Estimation Resource (TIMER) web server is an interactive database that helps comprehensively analyze immune cell infiltration (B cells, CD4+ T cells, CD8+ T cells, neutrophils, macrophages, and dendritic cells) in difference cancer types (https://cistrome.shinyapps.io/timer/). We applied ‘‘Gene’’ module of TIMER to evaluate the correlation between KIF15 expression and the six immune cell subtypes from the expression file.

TMB is defined as the total count of somatic insertions, base substitutions, and deletions in each coding area of the tumor genome. The Perl language and R software (version 4.1.1) were used to calculate the total TMB score of each TCGA cancer case and analyze its relationship with KIF15 expression level in pan-cancer (15). MSI is defined as the number of insertion or deletion events in short tandem repeat DNA tracts. Analysis of the correlation between KIF15 expression and MSI was performed by R software (16). Relationship between KIF15 expression and immune signatures was investigated. These immune signatures contained BTLA, CD200, TNFRSF14, NRP1, LAIR1, TNFSF4, CD244, LAG3, ICOS, CD40LG, CTLA4, CD48, CD28, CD200R1, HAVCR2, ADORA2A, CD276, KIR3DL1, CD80, PDCD1, LGALS9, CD160, TNFSF14, IDO2, ICOSLG, TMIGD2, VTCN1, IDO1, PDCD1LG2, HHLA2, TNFSF18, BTNL2, CD70, TNFSF9, TNFRSF8, CD27, TNFRSF25, VSIR, TNFRSF4, CD40, TNFRSF18, TNFSF15, TIGIT, CD274, CD86, CD44 and TNFRSF9, according to previous reports (15–17).The limma package and RColorBrewer package of R software were used to evaluate the correlation between KIF15 expression and the selected immunologic genes in pan-cancer.

### Genetic Alteration Analysis of KIF15

cBioPortal for Cancer Genomics database (http://cbioportal.org) were utilized to analysis the KIF15 alteration frequency, copy number alteration and mutation type in various cancer types from TCGA.

### GEO Database Analysis of NPC

Three gene expression profiling datasets GSE12452, GSE53819, and GSE61218 were obtained from the GEO database. The GSE12452 microarray contained 31 NPC samples and 10 normal samples, the GSE53819 microarray included 18 NPC samples and 18 non-cancerous samples, the GSE61218 microarray included 10 NPC tissue samples and six normal samples. The expression level of KIF15 was evaluated by wilcoxon, and *P*-values <0.05 were set as statistically significant.

### Immunohistochemistry and Evaluation

From April 2011 to December 2015, 158 formalin-fixed, paraffin-embedded NPC and 33 normal nasopharyngeal epithelium (NNE) tissues were collected in The First Affiliated Hospital of Guangxi Medical University. The patient tumors were newly diagnosed, non-metastatic, measurable, and pathologically confirmed to be NPC. The study was approved by the ethics committee of The First Affiliated Hospital of Guangxi Medical University.

First, the paraffin-embedded tissue sections were dewavered and rehydrated, then the antigen retrieval was carried out, and the endogenous peroxidase activity was blocked by 3% hydrogen peroxide for 25 min at 25°C. After being incubated with KIF15 primary antibody (Abcam, 1:200) at 4°C overnight, the sections were incubated by the secondary antibody for 90 min at room temperature. The immunoreactive score was calculated by multiplying the proportion of positive cells and the staining intensity. The cell positivity scores were determined as follows: <5% for zero; 5%–25% for one; 26%–50% for two; 51%–75% for three; and 76%–100% for four. The staining intensity scores were determined according to the following: 0 for no staining; 1 for light yellow; 2 for yellow; and 3 for brown. The final immunoreactive score were determined according to the following: 0 for negative, 1-3 for weak staining, 4-7 for moderate staining and, 8-12 for intense staining. All the NPC patients were divided into KIF15 high expression group and low expression group base on median immunoreactive score.

### Gene Set Enrichment Analysis (GSEA) of KIF15

GSEA was conducted using GSEA (version 4.0.1) with the Molecular Signatures Database (MSigDB). Samples were separated into high or low KIF15 expression groups based on the median KIF15 expression. The gene set ‘‘c2.cp.kegg.v7.1.symbols.gmt’’ of MSigDB gene set was chose as a reference gene set. A pathway with adj P-value<0.05, false discovery rate (FDR)<0.25 and normalized enrichment score (NES) >1.5 was considered as significantly enriched.

### Cell Lines and Transfection

The normal human nasopharyngeal epithelial cell line (NP69) and NPC cell line (CNE1, CNE2, HONE1, C666-1) were obtained from Guangxi Medical University Nasopharyngeal Cancer Research Laboratory. The NP69 cells were cultured in keratinocyte-SFM medium (Invitrogen, Carlsbad, USA) containing bovine pituitary extract (BD Biosciences, San Diego, CA, USA). Human NPC cells were cultured in RPMI-1640 medium supplemented with 10% fetal bovine serum (Gibco), 1% streptomycin/penicillin was added to the medium. All the cells were incubated in a humidified atmosphere with 5% CO_2_ at 37°C. NPC cell was transfected with siRNAs targeting KIF15 or control siRNA (RuiSai, Shanghai, China) using Lipofectamine 3000 (Invitrogen, Carlsbad, USA). The target sequences were GCGGTTATAATGGTACCAT (siKIF15-1), and GCTGGAAAGAGTTTCCTTT (siKIF15-2).

### Quantitative Real Time Polymerase Chain Reaction (qRT-PCR)

Total RNA from NPC cell was extracted using TRIzol reagent (Life Technologies Corporation, Carlsbad, USA), cDNA was generated using the PrimeScript RT reagent kit according to the manufacturer’s protocol (Takara Bio, Kusatsu, Japan). Then, the TB Green Premix Ex Taq II kit (Takara Bio) was applied for qRT-PCR. The relative RNA expression was determined by 2 ^-△△ct^ method, with GAPDH being the internal control. The primers sequences were as follows: KIF15: Forward: 5’-TGGAGGATGGAGGAATAG-3’; Reverse: 3’-CCACCAGGTTGAGTAGGG-5’. GAPDH: Forward: 5’-GGATTGTCTGGCAGTAGCC-3’; Reverse: 3’-ATTGTGAAAGGCAGGGAG-5’.

### Cell Viability and Colony Formation Assays

Cells were planted into 96 well plates (1500 cells/well) after 24 hours of transfection. CCK-8 reaction reagent (Dojindo, Japan) was used to measure cell viability at 0h, 24h, 48h, 72h. 10 µl of CCK-8 solution was added into each well and incubated for 2 h. The OD value was measured with the microplate reader at 450 nm. In order to explore proliferation, colony formation assay was performed. After incubation in 6-well plates at 1500/well, the formation of cell colonies was detected after 14 days. In brief, cells were subjected to methanol fixation and stained by crystal violet solution. clones contained at least 50 cells were counted for analysis.

### Scratch Assay

5 × 10^5^ cells/well were seeded into 6-well plates. Subsequently, 10µl pipette tip was used to create a wound on the confluent cell monolayer. Then, we used inverted microscope to take photos of wound closure at 0 and 24 h and the wound healing distance was analyzed.

### Transwell Assay

After resuspending by serum-free medium, 5 × 10^4^ cells containing RPMI 1640 medium without FBS were plated in the upper chamber, and 500 ul of 10% FBS RPMI 1640 medium was added to the lower chamber. The number of cells that had migrated after 24 h was measured under three random fields.

### Statistical Analysis

Differences in clinical characteristics (Gender, Age, Histological type, T stage, N stage and TNM stage) between the groups were evaluated using the chi square test, while 5 year-OS between the groups were evaluated using KM analysis with the log-rank test. OS, RFS, and DMFS were defined as the period from the day of first treatment to day of death, relapse and distant metastasis due to any reason. Statistical analysis and visualization were conducted with SPSS (version 24.0; IBM, New York, USA), GraphPad Prism (version 8.0), and R software. Results with *P*<0.05 was regarded as statistically significant. We conducted univariate and multivariate cox regression analyses for the selection of features. The selection of candidate features depended on comprehensive consideration of their clinical value and statistical significance. The nomogram model was generated with 5-year OS endpoint by the rms package of R software. Concordance index (C-index), receiver operating characteristic (ROC) curve, and the calibration curve were used to evaluate the predictive accuracy for the nomogram. After calculating the total scores by nomogram, patients were divided into low- or high-risk subgroups by using the X-tile software (version 3.6.1; Yale University, New Haven, CT, USA) (18).

## Results

### KIF15 mRNA Expression and Clinical Association in Pan-Cancer

**Figure 1:**
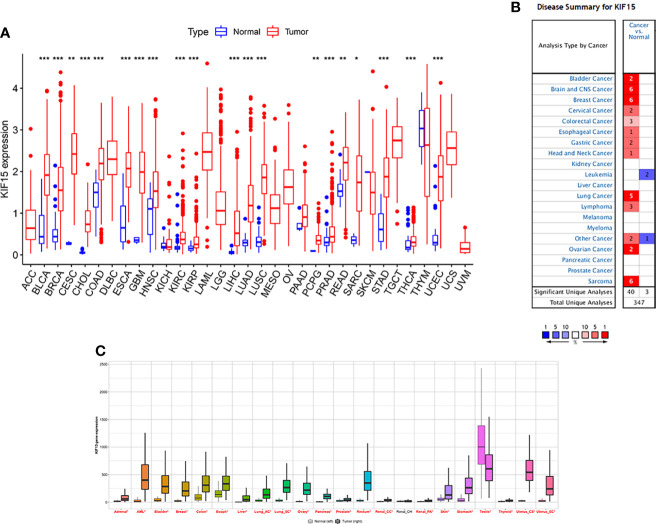
The expression level of KIF15 in pan-cancer. **(A)** Differential expression of KIF15 between tumor and normal tissues of KIF15 in TCGA. **(B)** Differential expression of KIF15 between tumor and normal tissues of KIF15 in Oncomine. **(C)** Differential expression of KIF15 between tumor and normal tissues of KIF15 in Kaplan–Meier plotter. *P < 0.05; **P < 0.01 and ***P < 0.001.

**Figure 2:**
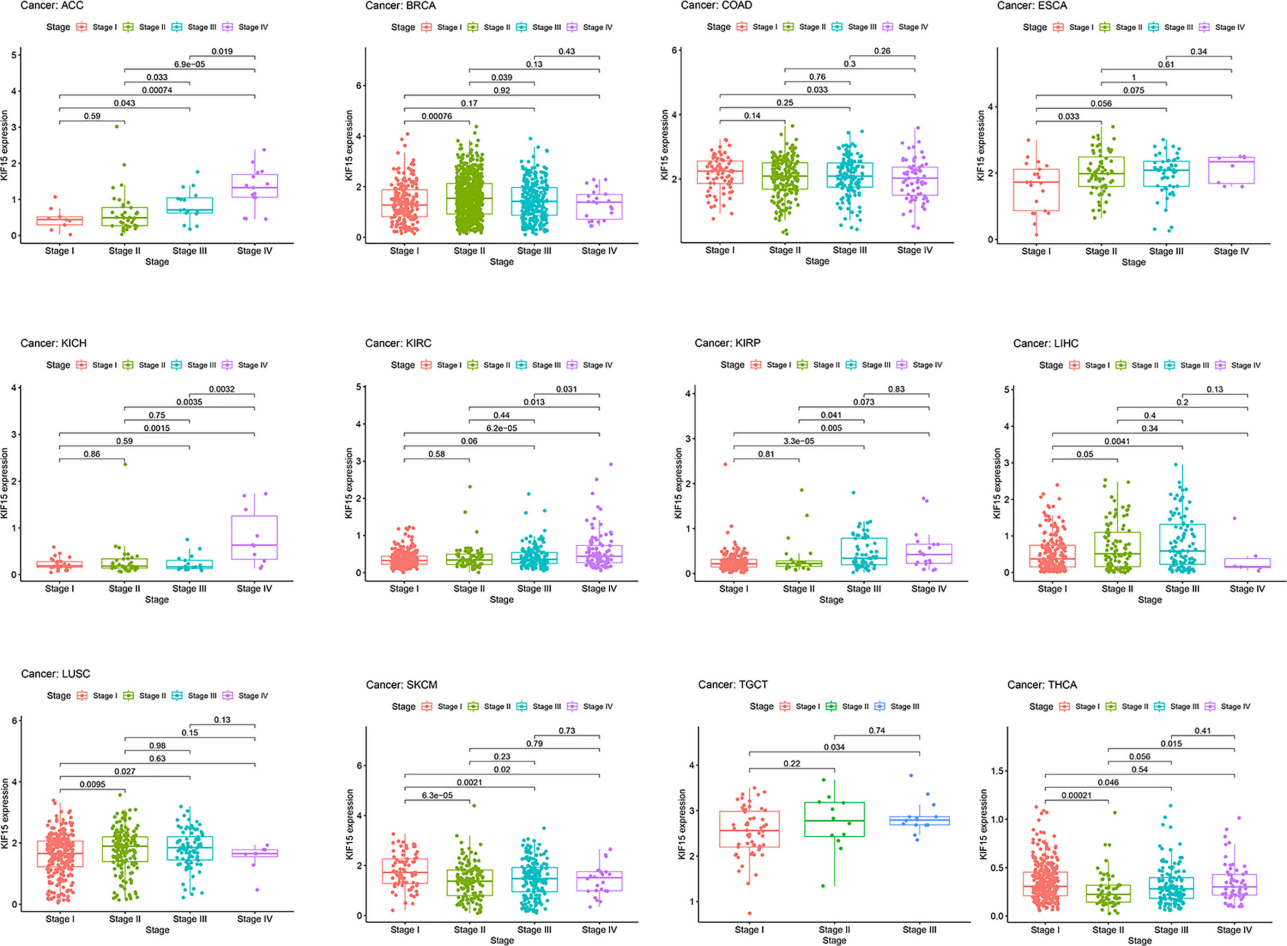
Correlation of KIF15 mRNA expression and different pathological stages of certain cancers in TCGA.

The abbreviations of the 33 TCGA cancer types are shown in **Table 1**. In TCGA database, KIF15 was upregulated in 20 cancer types, including BLCA, BRCA, CESC, CHOL, COAD, ESCA, GBM, HNSC, KICH, KIRC, LIHC, LUAD, LUSC, PCPG, PRAD, READ, SARC, STAD, THCA and UCEC (**Figure 1A**). Likewise, in the Oncomine database, the expression level of KIF15 was significantly increased in bladder, brain and CNS, breast, cervical, colorectal, esophageal, gastric, head and neck, lung, and ovarian cancers, as well as lymphoma and sarcoma; while significantly decreased in leukemia. (**Figure 1B**). Detailed data of KIF15 expression levels in Oncomine database are shown in **Supplementary Table 1**. In the Kaplan-Meier plotter database, KIF15 was differentially highly expressed in 18 cancer types, including adrenal, bladder, breast, colorectal, esophageal, liver, lung, ovarian, pancreatic, prostate, rectal, renal, skin cancer, stomach, thyroid, and uterine cancers and acute myeloid leukemia, while less expressed in testicular cancer (**Figure 1C**). In short, KIF15 could serve as an oncogene in pan-cancer. In addition, the expression level of KIF15 significantly related to patients’ cancer stage in ACC, BRCA, COAD, ESCA, KICH, KIRC, KIRP, LIHC, LUSC, SKCM, TGCT, and THCA based on TCGA database (**Figure 2**).

**Table 1:** Abbreviations of the 33 cancer types in the The Cancer Genome Atlas database.

ACC	Adrenocortical carcinoma
BLCA	Bladder urothelial carcinoma
BRCA	Breast invasive carcinoma
CESC	Cervical squamous cell carcinoma and Endocervical adenocarcinoma
CHOL	Cholangiocarcinoma
COAD	Colon adenocarcinoma
DLBC	Lymphoid neoplasm diffuse large B-cell lymphoma
ESCA	Esophageal carcinoma
GBM	Glioblastoma multiforme
HNSC	Head and Neck squamous cell carcinoma
KICH	Kidney chromophobe
KIRC	Kidney renal clear cell carcinoma
KIRP	Kidney renal papillary cell carcinoma
LAML	Acute myeloid leukemia
LGG	Brain lower grade glioma
LIHC	Liver hepatocellular carcinoma
LUAD	Lung adenocarcinoma
LUSC	Lung squamous cell carcinoma
MESO	Mesothelioma
OV	Ovarian serous cystadenocarcinoma
PAAD	Pancreatic adenocarcinoma
PCPG	Pheochromocytoma and Paraganglioma
PRAD	Prostate adenocarcinoma
READ	Rectum adenocarcinoma
SARC	Sarcoma
SKCM	Skin cutaneous melanoma
STAD	Stomach adenocarcinoma
TGCT	Testicular germ cell tumors
THCA	Thyroid carcinoma
THYM	Thymoma
UCEC	Uterine corpus endometrial carcinoma
UCS	Uterine carcinosarcoma
UVM	Uveal melanoma

### Correlation Analysis Between the Expression of KIF15 and Prognostic Value

**Figure 3:**
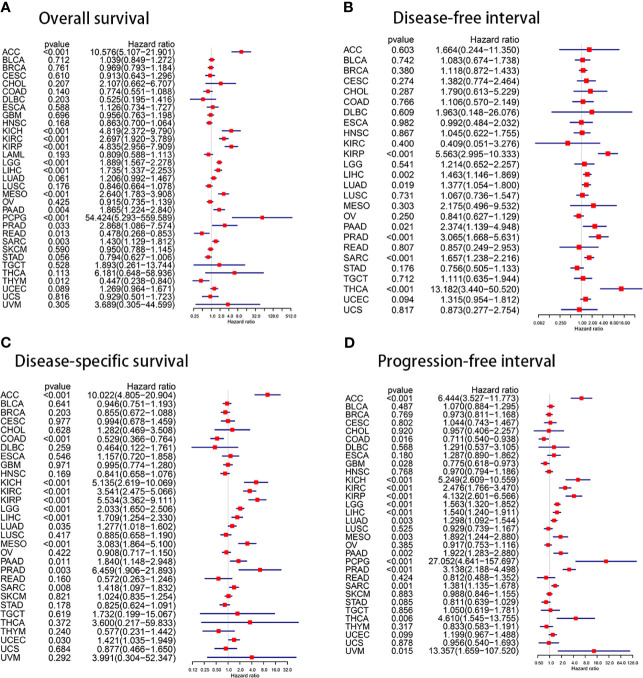
The prognosis value of KIF15 of differ cancers using Univariate Cox proportional hazards models. **(A)** Overall survival (OS). **(B)** Disease-free survival (DFI). **(C)** Disease-specific survival (DSS). **(D)** Progression-free interval (PFI).

Univariate cox regression analyses are shown as forest charts in **Figure 3**. High KIF15 expression positively correlated with poorer OS in ACC, KICH, KIRC, KIRP, LGG, LIHC, MESO, PAAD, PCPG, PRAD, READ, while it correlated with better OS in READ and THYM (**Figure 3A**). For DFI, high KIF15 expression remarkably correlated with worse survival in KIRP, LIHC, LUAD, PAAD, PRAD, SARC, and THCA (**Figure 3B**). For DSS, it was found that high KIF15 expression significantly correlated with worse prognosis in ACC, KICH, KIRC, KIRP, LGG, LIHC, LUAD, MESO, PAAD, PRAD, SARC, and UCEC, while it correlated with better prognosis in COAD (**Figure 3C**). For PFI, high KIF15 expression positively correlated with worse survival in ACC, KICH, KIRC, KIRP, LGG, LIHC, LUAD, MESO, PAAD, PCPG, PRAD, SARC but correlated with better survival in COAD and GBM (**Figure 3D**).

**Figure 4:**
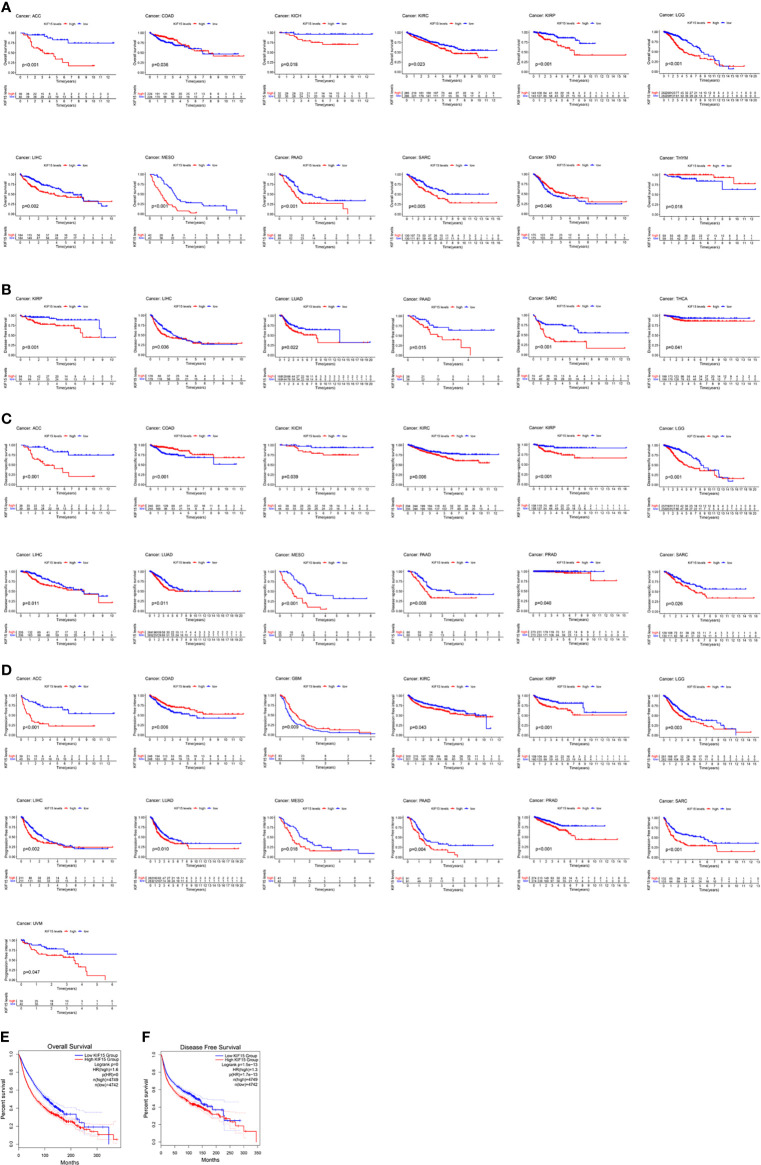
The prognostic value of KIF15 in different cancers using Kaplan–Meier method. **(A)** Overall survival (OS). **(B)** Disease-free survival (DFI). **(C)** Disease-specific survival (DSS). **(D)** Progression-free interval (PFI). **(E)** OS curve of KIF15 in all cancer based on GEPIA. **(F)** Disease-free survival (DFS) curve of KIF15 in all cancer based on GEPIA.

A K-M survival curve was used to demonstrate the effect of KIF15 on prognosis, as shown in **Figure 4**. For OS, increased KIF15 expression showed worse prognosis in ACC, KICH, KIRC, KIRP, LGG, LIHC, MESO, PAAD, and SARC but better prognosis in COAD, STAD, and THYM (**Figure 4A**). For DFI, increased KIF15 expression showed worse prognosis in KIRP, LIHC, LUAD, PAAD, SARC, and THCA (**Figure 4B**). For DSS, increased KIF15 expression showed worse prognosis in ACC, KICH, KIRC, KIRP, LGG, LIHC, LUAD, MESO, PAAD, PRAD and SARC, but better prognosis in COAD (**Figure 4C**). For PFI, increased KIF15 expression showed worse prognosis in ACC, KIRC, KIRP, LGG, LIHC, LUAD, MESO, PAAD, PRAD, SARC, and UVM but better prognosis in COAD and GBM (**Figure 4D**). Together, higher expression levels of KIF15 represented an unfavorable prognostic indicator in pan-cancer. Additionally, based on the GEPIA platform, higher mRNA expression levels of KIF15 also indicated a worse prognostic outcome in pan-cancer (**Figures 4E, F**).

A meta-analysis was performed to further clarify the prognostic value of KIF15 in cancers. The flowchart of literature retrieval process is shown in **Supplementary Figure 1**. The basic characteristics of all included studies (12, 14, 19–29) or datasets are shown in **Supplementary Table 2** and **Supplementary Table 3**. Results of the forest plots demonstrated that high KIF15 expression significantly correlated to a worse OS (HR 1.25, 95% CI 1.14–1.37, *P*<0.0001); RFS (HR 1.31, 95% CI 1.13–1.53, *P* = 0.0003); and DMFS (HR 1.51, 95% CI 1.32–1.73, *P*<0.00001) (**Supplementary Figure 2**). Significant heterogeneity in meta-analysis was observed (OS, *P*<0.00001, *I* ^2^ = 60%; RFS, *P* = 0.01, *I* ^2^ = 53%), and thus a REM was adopted. For the sensitivity analysis of OS, after exclusion of Liu et al., Song et al., and Duke OC, the heterogeneity was reduced (*P*=0.0003, *I* ^2 ^= 48%), while no significant change occurred with the HR of 1.30 (95% CI 1.19–1.43, *P*<0.00001). For the sensitivity analysis of RFS, after exclusion of GSE31210, the heterogeneity decreased (*P*=0.23*, I* ^2 ^= 22%), while the HR slightly decreased to 1.23 (95% CI 1.09–1.38, *P*=0.0006) (**Supplementary Figure 3**). Thus, the summarized results in the meta-analysis were relatively reliable and stable. In summary, these integrated analyses suggest that high expression of KIF15 may serve as a poor prognostic biomarker in most cancers.

### Molecular Mechanism of KIF15 in Pan-Cancer

**Figure 5:**
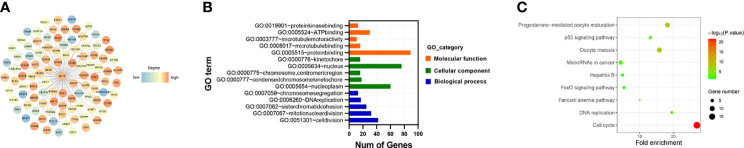
Functional analysis of KIF15 and relevant genes. **(A)** Protein–protein interaction (PPI) network display the top 100 relevant genes of KIF15. **(B)** Gene Oncology (GO) analysis of KIF15 and relevant genes. **(C)** Kyoto Encyclopedia of Genes and Genomes (KEGG) analysis of KIF15 and relevant genes.

The interaction between KIF15 and its related genes are displayed in **Figure 5A**. KEGG and GO enrichment analysis were carried out to explore the potential functions of KIF15 in cancer. The results indicated that KIF15 and its related genes were significantly associated with cell division, mitotic nuclear division, and sister chromatid cohesion; they may also have association with the p53 signaling pathway, the cell cycle, and DNA replication (**Figures 5B, C**). In the analysis of CancerSEA database, the functional state of KIF15 was explored at the single-cell level in 14 types of cancer. KIF15 was found to be positively associated with cell cycle, DNA damage, DNA repair, and proliferation in multiple cancer types (**Supplementary Figure 4**).

### Genetic Alteration of KIF15 in Pancancer

**Figure 6:**
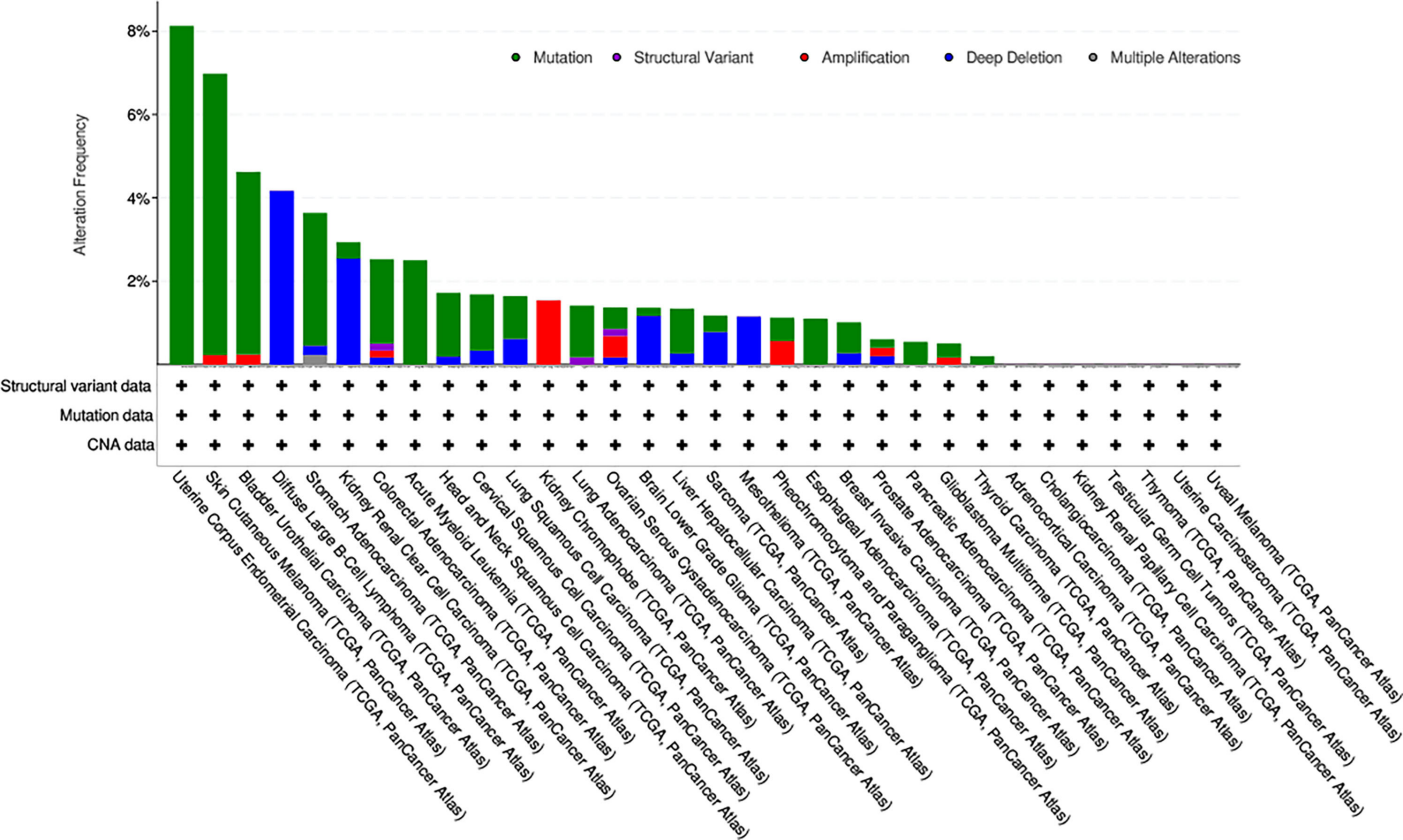
The alteration frequency with mutation type of KIF15 in different cancer types from TCGA database.

As is shown in **Figure 6**. Mutation status of KIF15 was evaluated, the highest alteration rate of KIF15 (8.13%) appears in patients with uterine corpus endometrial carcinoma with ‘‘mutation’’ as the primary type. The ‘‘deep deletion’’ type (4.17%) of copy number alteration was the primary type in the diffuse large B-Cell lymphoma cases.

### Relationship Between KIF15 and Immune-Related Factors

**Figure 7:**
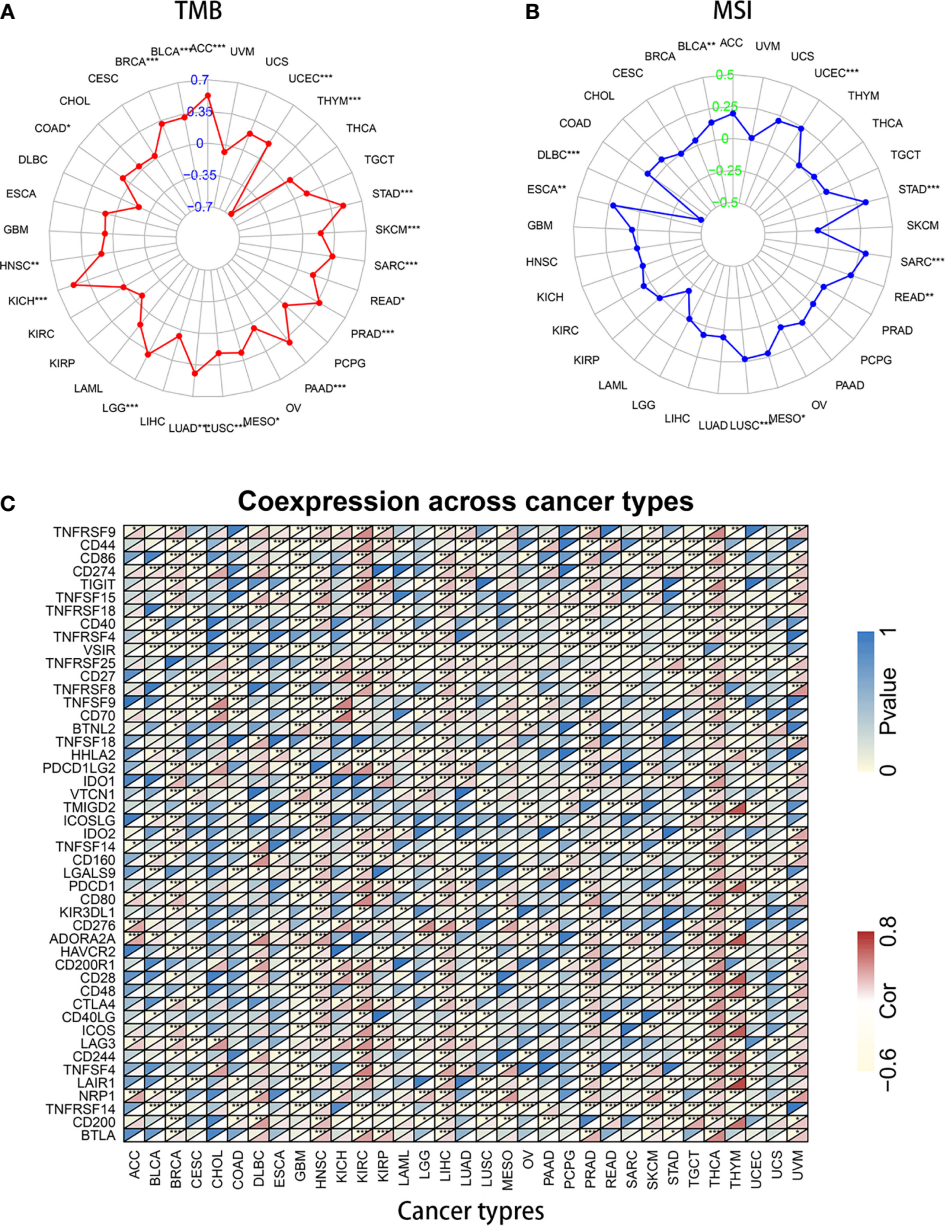
Association of KIF15 mRNA expression with tumor mutational burden (TMB), microsatellite instability (MSI) and immune genes. **(A)** A radar map shows the relationship of KIF15 and TMB. **(B)** A radar map shows the relationship of KIF15 and MSI. **(C)** Heatmap shows the relationship of KIF15 and immune genes. *P < 0.05; **P < 0.01 and ***P < 0.001.

Studies have proven that immune cell infiltration is significantly correlated with survival in cancers. Tumor purity is a vital factor that affects the evaluation of immune infiltration. Therefore, the relationship between KIF15 expression and immune cell infiltration in pan-cancer was explored. Notably, the results indicated that HNSC, KIRC, LGG, LIHC, PRAD, and THYM were six cancer types most strongly associated with KIF15 expression in immune cell infiltrating level, including B cells, CD8+ T cells, CD4+ T cells, macrophages, neutrophils, and dendritic cells (**Supplementary Figure 5**). TMB and MSI were also analyzed. For TMB, it was found that KIF15 gene expression was positively related to ACC, BLCA, BRCA, COAD, HNSC, KICH, LGG, LUAD, LUSC, MESO, PAAD, PRAD, READ, SARC, SKCM, STAD, and UCEC but negatively related to THYM. For MSI, we found that KIF15 gene expression was positively related to BLCA, ESCA, LUSC, MESO, READ, SARC, STAD, and UCEC but was negatively related to DLBC. Moreover, correlation between KIF15 and immune gene set was analyzed, and the expression of several important immune-related genes was significantly related to KIF15 expression level in pan-cancer, such as CTLA4, IDO1, and LAG3 (**Figure 7**). In summary, our findings showed that high expression of KIF15 played an important role in immune-related factors.

### KIF15 Expression in NPC Tissue

**Figure 8:**
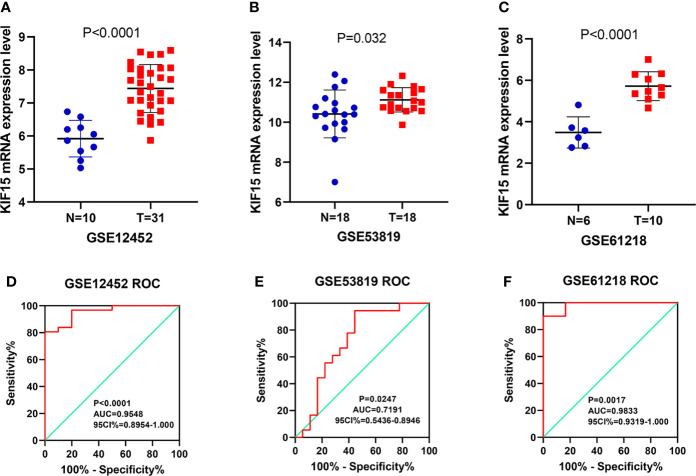
The expression of KIF15 in nasopharyngeal carcinoma (NPC) and normal tissues were investigated by GEO database. **(A–C)** KIF15 was significantly upregulated in NPC tissue in three datasets. **(D–F)** The diagnostic operating characteristic (ROC) curves of KIF15 in NPC and normal tissues in three datasets.

**Figure 9:**
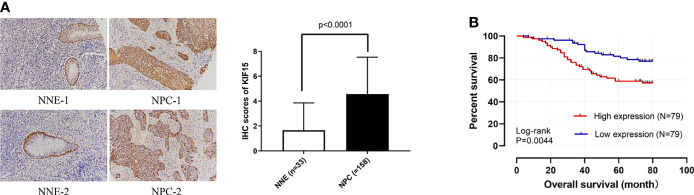
Preliminary experimental verification of KIF15 in patients with nasopharyngeal carcinoma (NPC). **(A)** Immunohistochemical analysis of KIF15 protein expression between nasopharyngeal carcinoma (NPC) and normal tissues (200×). **(B)** Kaplan–Meier survival curves for KIF15 in NPC.

**Figure 10:**
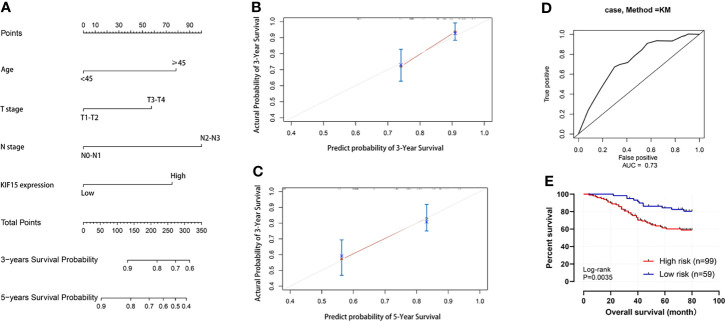
Nomogram for predicting the prognosis of NPC patient. **(A)** Nomogram. **(B, C)** 3-year and 5-year calibration curves. **(D)** Operating characteristic (ROC) curves for the mode. **(E)** Survival curve of high-risk and low-risk groups.

Regarding the key functional role of KIF15 in cancers, we detected the expression level of KIF15 in NPC tissue through the GEO datasets and IHC. Based on the analyses of three microarrays (GSE12452, GSE53819, and GSE61218), KIF15 expression was found to be upregulated in NPC tissues compared to the normal controls (*P*<0.05) (**Figures 8A–C**). A diagnostic ROC curve was performed between the two groups, and KIF15 exhibited high diagnostic value in the three microarrays (area under the curve, AUC= 0.9584, 0.7191, and 0.9833, respectively) (**Figures 8D–F**). For IHC, Compared with the NNE tissue, KIF15 expression was significantly upregulated in NPC tissues (**Figure 9A**). As shown in **Table 2**, high KIF15 expression level positively correlated with T stage (*P*=0.015), N stage (*P*=0.003), and clinical stage (*P*=0.006). the median follow-up period was 76 months (range, 4-80 months). After 5-year follow-up, 62 (78.5%) patients in low KIF expression group were alive, 46 (58.2%) patients in low KIF expression group were alive. The result indicated that increased KIF15 expression correlated with worse OS for NPC (P=0.0044). (**Figure 9B**). To identify the independent prognostic factor in NPC, univariate analysis was used to assess the prognostic value of clinical features and KIF expression level. These results indicated that age (*P*=0.078), T stage (*P*=0.023), N stage (*P*=0.007), and KIF15 expression level (*P*=0.006) were significantly correlated with the OS (**Table 3**). According to the multivariate cox analysis, age, T stage, N stage, and KIF15 expression level were incorporated to build a nomogram model (**Figure 10A**). The C-index of the model was 0.695 (95% CI 0.62–0.765) and was verified by 1000-replication bootstrapping analysis. The calibration curves for predicting 3- and 5-year OS also indicated a satisfactory predictive accuracy (**Figures 10B, C**). ROC curve analysis revealed that the model had an effective predictive ability, with an AUC value of 0.730 (**Figure 10D**). Subsequently, based on the total score of each case, all the patients were divided into either a low-risk (score<136) or high-risk group by X-tile software. KM survival curves showed that the OS in the high-risk group was significantly reduced below that of the low-risk group (**Figure 10E**).

**Table 2:** Correlation between the expression level of KIF15 and clinicopathological characteristics of patients with nasopharyngeal carcinoma.

Variables	Case	KIF15 expression		
		low	high	*P-value*
Gender				
Female	39	17	22	
Male	119	62	57	0.356^†^
Age (y)				
< 45	73	35	38	
≥ 45	85	44	41	0.632^†^
Histological type				
WHO II	14	6	8	
WHO III	144	73	71	0.576^†^
T stage				
T1-2	65	40	25	
T3-4	93	39	54	0.015^†^
N stage				
N0-1	56	37	19	
N2-3	102	42	60	0.003^†^
TNM stage				
I-II	32	23	9	
III-IV	126	56	70	0.006^†^
5 year-OS		78.5%	58.2%	0.0044^‡^

OS, overall survival. ^†^chi square test, ^‡^log-rank test.

**Table 3:** Evaluation of the prognostic factors of nasopharyngeal carcinoma based on univariate and multivariate COX regression.

	Univariate Cox regression			Multivariate Cox regression		
	HR	95% CI	*P*-value	HR	95% CI	*P*-value
Gender (Female vs. Male)	0.683	0.341-1.365	0.280	–	*-*	*-*
Age (≥45 vs. <45)	1.681	0.943-2.99	0.078	1.957	1.092-3.506	0.024
T stage (T3–4 vs. T1–2)	2.019	1.102-3.699	0.023	1.637	0.885-3.028	0.116
N stage (N2–3 vs. N0–1)	2.590	1.294-5.180	0.007	2.355	1.155-4.801	0.018
TNM stage (III–IV vs. I–II)	1.815	0.816-4.036	0.144	–	–	–
Histological type (WHO III vs. WHO II)	1.626	0.506-5.224	0.415	–	–	–
KIF15 expression (High vs. Low)	2.284	1.272-4.104	0.006	1.902	1.047-3.454	0.035

HR, hazard ratio; CI, confidence interval.

### KIF15 Related Pathways

**Figure 11:**
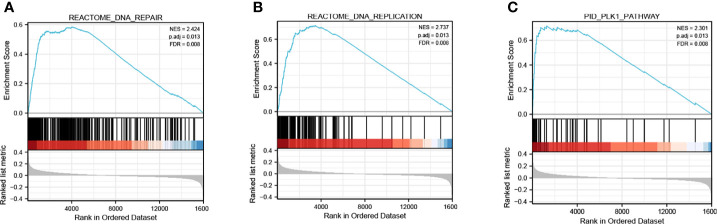
Enrichment plots of GSEA from GSE12452 dataset. **(A–C)** KIF15 related signaling pathways in c2.cp.kegg.v7.1.symbols.gmt.

As is shown in **Figure 11**, GSEA analysis of GSE12450 indicated that high expression of KIF15 significantly related to DNA repair (NES = 2.424, p.adj = 0.013, FDR = 0.008), DNA replication (NES = 2.737, p.adj = 0.013, FDR = 0.008) and PLK1 pathway (NES = 2.301, p.adj = 0.013, FDR = 0.008).

### Primary Validation of the Effect of KIF15 in NPC Cells

**Figure 12:**
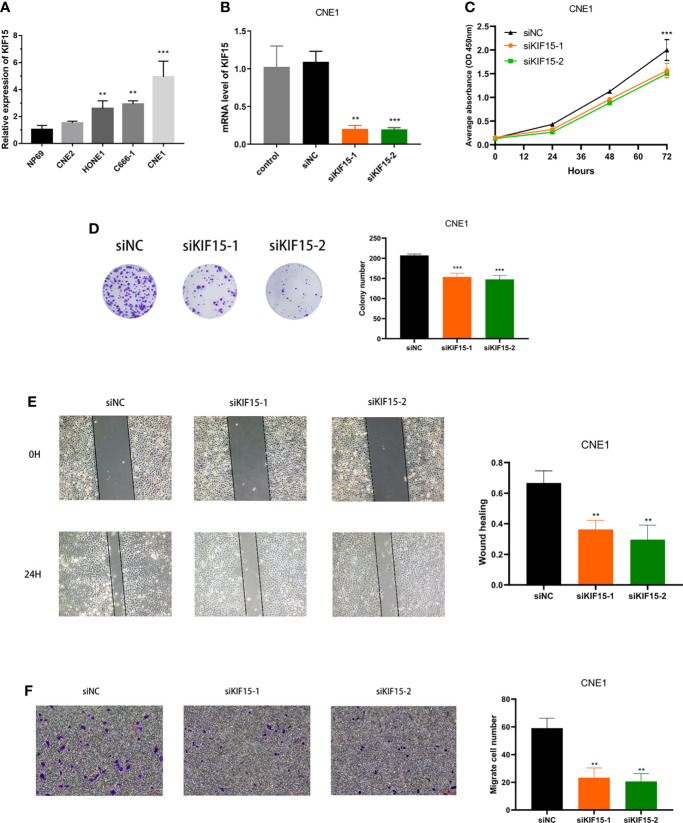
Effect of KIF15 in NPC cell proliferation and migration. **(A)** qRT-PCR analysis of KIF15 expression level in normal and NPC cell lines. **(B)** qRT-PCR analysis confirmed the knockdown efficacy of KIF15 in NPC cell line CNE1. **(C, D)** CCK-8 and colony formation assay were applied to examine the proliferation ability of KIF15 knockdown cells. **(E, F)** Wound-healing and transwell assay employed to detect the migration ability of KIF15 knockdown cells. **P < 0.01 and ***P < 0.001.

Using RT-PCR, we found that the mRNA expression level of KIF15 was increased in NPC cell lines, especially in CNE1, compared to the NP69 (p < 0.001) (**Figure 12A**). Therefore, CNE1 cell line was selected for further study. Besides, We verified that KIF15 expression level was significantly repressed upon si-KIF15 transfection (**Figure 12B**). CCK8 and colony formation assays indicated that downregulation of KIF15 remarkably reduced the proliferation of CNE1 (**Figures 12C, D**). Wound healing was remarkedly suppressed by KIF15 silencing in CNE1 (**Figure 12E**). The Transwell assay indicated that the migration of CNE1 cells were significantly suppressed by KIF15 silencing (**Figure 12F**).

## Discussion

NPC, one of the major types of head and neck cancer, is a malignant tumor arising from the nasopharyngeal mucosal lining (30). Because of its challenging anatomical location, radiochemotherapy is regarded as the mainstay of treatment (31). However, an anatomy-based staging system is not enough to predict prognosis and treatment efficiency of NPC. Thus, it is necessary to investigate the incorporation of clinical features and novel biomarkers for the improvement of the efficacy of prediction.

Kinesins are a type of conserved protein that modulate the movement of certain important functional molecules, including chromosomes, protein complexes, mRNAs, and organelles in cells during mitosis (32). Thus, they are critical for protein sorting and appropriate positioning of different biological molecules. Reportedly, the kinesin family features are prominent in facilitating a variety of biological processes such as cell morphology, cytoskeletal dynamics, cell division, and cell migration (8). These findings have demonstrated a very promising role of the kinesin family in cancer. For instance, KIF23 is highly expressed in gastric cancer and is correlated with a worse prognosis of patients (33). KIF21B has been identified as an oncogene in the development and migration of NSCLC (34). Additionally, KIF20B could promote cancer growth by promoting cell proliferation in tongue cancer (35). However, the effect of most kinesin families in tumorigenesis are not completely understood.

KIF15 is a member of the kinesin family that directs kinesin-like motor enzymes involved in mitotic spindle assembly (36). The aberrant expression of KIF15 could lead to abnormal cell replication, differentiation, and thus cause tumorigenesis. The expression pattern and functional roles of KIF15 in tumor pathogenesis, especially in NPC, have not been comprehensively investigated. The results of our pan-cancer analysis revealed that KIF15 expression was significantly upregulated in most type of cancers, suggesting that KIF15 might act as an oncogene in pan-cancers. Based on KM and univariate analyses, it was found that high expression of KIF15 indicated poor survival in several cancers, including OS, DFI, DSS, and PFI. Our results from the clinical correlation test showed that KIF15 expression level was increased in advanced pathological stages in ACC, KICH, KIRC, KIRP, LIHC, LUAD, and TGCT. Moreover, the results of IHC revealed that KIF15 had a higher expression level in NPC tissues, and it was significantly correlated with the poor prognosis of patients with NPC. Previous studies have indicated that KIF15 plays a vital role in the disease progression of most cancers. Gao et al. have found that the upregulation of KIF15 in breast cancer tissues was positively related to TNM stage, tumor size, and lymph node metastasis; while downregulation of KIF15 inhibited cell proliferation and tumor proliferation *in vitro* and *in vivo* (37). Research by Li et al. has shown that over-expression of KIF15 promoted the cancer stem cell (CSC) phenotype and malignancy through phosphoglycerate dehydrogenase (PHGDH)-regulated intracellular reactive oxygen species disorders in HCC (38). Wang et al. have also shown that KIF15 promoted pancreatic cancer growth by enhancing G1/S phase transition by affecting the MEK–ERK signalling pathway (25). These findings are consistent with that of ours, which indicated that KIF15 is a promising diagnostic and prognostic biomarker in pan-cancer, as well as NPC.

Functional enrichment analysis showed that KIF15 may be involved in the p53 signaling pathway, the cell cycle, DNA replication and FOXO pathway. The well-known cancer suppressor gene, p53, closely controls various cellular signals involved in the cell-cycle, apoptosis, and senescence (39). A study based on machine learning showed that KIF20A and KIF23 were regulated by p53 and correlated with malignant transformation and tumor stage (40). Further, KIF15 knockdown strongly enhanced the expression of p53 and p21 protein in breast cancer cells (37). KIF15 is involved in Burkitt lymphoma cell activity *via* mediating the expression of p53 (41). Loss or mutation of p53 in tumor might have an impact on the recruitment and activity of myeloid and T cells, which contribute to immune evasion and tumor development, in addition, p53 can also affect the immune cells, causing different outcomes that can impede or promoting cancer progression (42, 43). The cell cycle is one of the most important topics studied in cancer biology. Over-expression of KIF15 increases the cyclin-D1, CDK2, p-RB expression, and accelerated G1/S transition in pancreatic cancer cells (25). KIF15 suppression has been shown to cause cell cycle arrest at the G0/G1 phase in breast cancer cells, indicating that knockdown of KIF15 inhibited the malignant behavior of breast cancer cells (27). Abnormal DNA replication is a hallmark of the cancer process, and previous studies have indicated that KIF15 and other kinesin genes were significantly enriched in DNA replication in bladder and endometrial cancers (29, 44). Accumulating evidence revealed that the FOXO family of transcription factors plays an important role in regulating the progression and function of tumor microenvironment (TME). FOXOs promote antitumor activity by negatively inducing the expression of immunosuppressive proteins, including PD-L1 and VEGF in stromal cells or tumor cell, and thus promote immunotolerant state in the TME (45).

TMB is an emerging characteristic of cancer and is tightly associated with MSI (46). Both TMB and MSI are considered to be biomarkers for the favorable immune checkpoint blockade treatment response in cancer (47, 48). For TMB, we found that KIF15 gene expression was positively associated with ACC, BLCA, BRCA, COAD, HNSC, KICH, LGG, LUAD, LUSC, MESO, PAAD, PRAD, READ, SARC, SKCM, STAD, and UCEC but was negatively associated with THYM. We suspect that a high neoantigens load led to the dysregulation of KIF15, and thus affected the development of cancer. For MSI, we found that KIF15 gene expression was positively associated with BLCA, ESCA, LUSC, MESO, READ, SARC, STAD, UCEC but was negatively associated with DLBC, suggesting that MSI may change the expression of KIF15 (15). Several kinesin superfamily have been found to link with tumor immune cells infiltration. For example, Ren et al. found that KIF20A expression was strong positive association with Th2 cells, Treg cells and Macrophages, while a negative association with Th17 cells, Mast cells and NK cells (49). Kim et al. indicated that KIF18A act as a key dendritic cells differentiation and activation regulator (50). Qiu et al. shown that KIF18B expression was associated closely with tumor immunity and interacted with various immune cells and genes markers (15). However, researches investigating the possible role of KIF15 in the regulation of tumor immunity are seldom. A study constructed a decision tree using mutations in PIK3CA, MEF2C, SLC11A1, and KIF15 to divided patient sub-cohorts with elevated PD-L1 expression, which contribute to identify the novel prognostic biomarkers of Gastric Cancer (51). Result of our study indicates new antigen generation was related to KIF15, further experiments are needed to investigate the regulator role of KIF15 in TME.

Tumor cells as well as the tumor microenvironment (TME) could secrete or express different signaling molecules, which act on immune checkpoints expressed in immune cells to inhibit immune responses (52). Several kinesin family genes have been linked to immune infiltration. For example, KIF18B expression significantly correlated negatively with the purity of stromal cells and immune cells in seven types of cancer (15). Likewise, KIF1A expression negatively correlated with infiltration levels of 16 types of immune cells in ovarian carcinoma (53). KIF20A had a strong positive association with Th2 cells, Treg cells, and macrophages but a negative association with Th17 cells, mast cells, and NK cells in renal clear cell carcinoma (49). However, the effects of KIF15 in cancer immunity and cancer microenvironment have been seldomly reported, and further investigation is urgently needed to clarify its role in cancer. In our study, expression of KIF15 significantly related to B cell, CD8+ T cell, CD4+ T cell, macrophage, neutrophil, and dendritic cell infiltration in HNSC, KIRC, LGG, LIHC, PRAD, and THYM. Macrophages have high plasticity in response to different external signals and directly influence various steps in tumor development, such as tumor cell proliferation, stemness, and immunosuppression (54). Neutrophils participate in almost every step of oncogenesis, and in recent years, the neutrophil-to-lymphocyte ratio has been regarded as a prognostic indicator of worse OS in cancer (55). Dendritic cells are a critical factor in antitumor immunity due to their potent antigen-presenting ability, therefore, dendritic cells are a critical target in any effort to generate immunotherapy against cancer (56). In short, our results suggest a likely regulatory role of KIF15 in tumor immunology.

Moreover, we selected several common immune genes and examined their correlation with KIF15 expression levels in various cancer types. Among these genes, CTLA4 has presently garnered much attention. CTL-associated antigen 4 (CTLA4) is the first immune checkpoint receptor to be clinically targeted. It regulates T-cell activation by competing with the co-stimulatory molecule CD28, CTLA4 and CD28 shared ligands, CD80 (also known as B7.1), and CD86 (also known as B7.2) (57). Once antigen recognition has started, CD28 signalling intensely amplifies TCR signalling to activate T cells (58). In this study, KIF15 significantly related to CTLA4 expression in 17 out of 33 cancer types. Indoleamine 2, 3-dioxygenase 1 (IDO1) is a novel immune checkpoint target, which is a type of a rate-limiting metabolic enzyme that transforms tryptophan (Trp) into downstream kynurenines (Kyn). Some studies have demonstrated that IDO1 was associated with potently regulating immunosuppressive effects in cancer (59). According to our findings, KIF15 was highly related to IDO1 expression in 11 out of 33 cancer types. Inhibitory receptors (IRs) have a potential role in regulating the immune response and are regulators of T cell dysfunction in autoimmune diseases. Lymphocyte Activation Gene 3 (LAG3), also known as CD223, is currently one of the most promising new IR targets in the clinic. It is expressed by both activated and exhausted CD4+ and CD8+ T cells as well as by regulatory T cells (60). In the present study, it was found that KIF15 was closely related to the expression of LAG3 in 16 out of 33 cancer types. Thus, KIF15 might serve as novel cancer therapeutic targets.

To the best of our knowledge, this is the first study that focused on the value of KIF15 from a pan-cancer perspective. We successfully explored the role of KIF15 in NPC; however, further functional experiments are still needed to clarify its effect on tumor biological process *in vivo* and *in vitro*. Despite the limitation of our study, we conclude that KIF15 could be a promising prognostic biomarker in pan-cancer as well as in NPC.

## Conclusion

In the present study, a pan-cancer investigation was performed revealing that KIF15 played a vital role in prognosis, molecular function, signaling pathways, and tumor immunity in differ cancer types based on public databases. Furthermore, we demonstrated that KIF15 was highly expressed in NPC tissue and could be considered as a novel diagnostic and prognostic biomarker of NPC.

## Data Availability Statement

The original contributions presented in the study are included in the article/**Supplementary Material**. Further inquiries can be directed to the corresponding authors.

## Ethics Statement

Written informed consent was obtained from the individual(s) for the publication of any potentially identifiable images or data included in this article.

## Author Contributions

LJ and RW conceived and designed the study. JM, SM, and WC organize and carried out the research. MK, MX, CL, BL, and FW helped to analyze the data of the study. FL and YZ performed the experiments. JM wrote the paper. All authors contributed to the article and approved the submitted version.

## Funding

This work was supported by the Guangxi Medical and Health Appropriate Technology Development and Promotion Application Project (No. S2017020), the Key Research and Development Program of Guangxi (No. Guike AB18281003), the “139” Program for high-level medical talents in Guangxi, Innovation Team of The First Affiliated Hospital of Guangxi Medical University, Guangxi Science and Technology Program Project (GK AD17129013), and the National Natural Science Foundation of China (No. 82060019, 82060494).

## Conflict of Interest

The authors declare that the research was conducted in the absence of any commercial or financial relationships that could be construed as a potential conflict of interest.

## Publisher’s Note

All claims expressed in this article are solely those of the authors and do not necessarily represent those of their affiliated organizations, or those of the publisher, the editors and the reviewers. Any product that may be evaluated in this article, or claim that may be made by its manufacturer, is not guaranteed or endorsed by the publisher.
