# Supplementary figures and images for: Integrative Pan-Cancer Analysis of KIF15 Reveals Its Diagnosis and Prognosis Value in Nasopharyngeal Carcinoma
Source: Front Oncol. 2022 Mar 11;12:772816. doi: 10.3389/fonc.2022.772816 (PMC8963360; doi:10.3389/fonc.2022.772816)

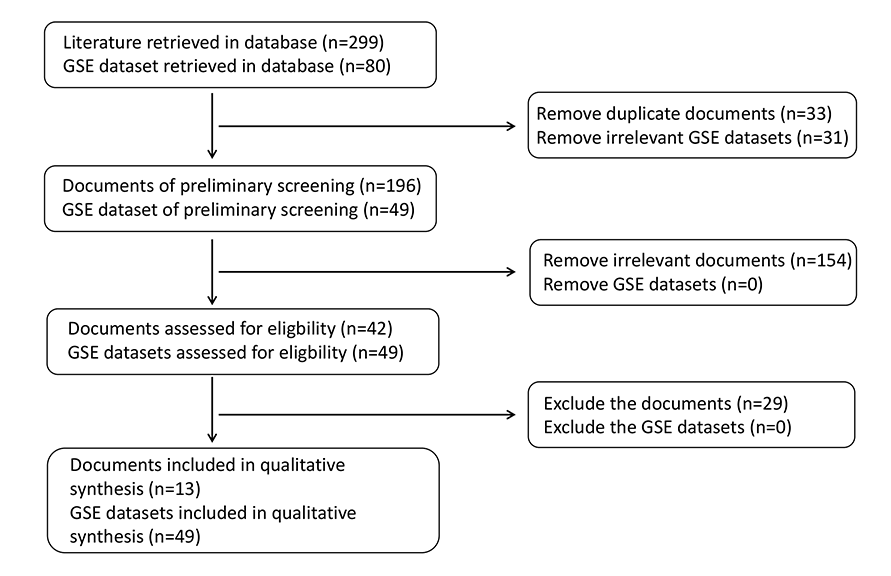

Supplement: Supplementary file 1 [file Image_1.tiff]

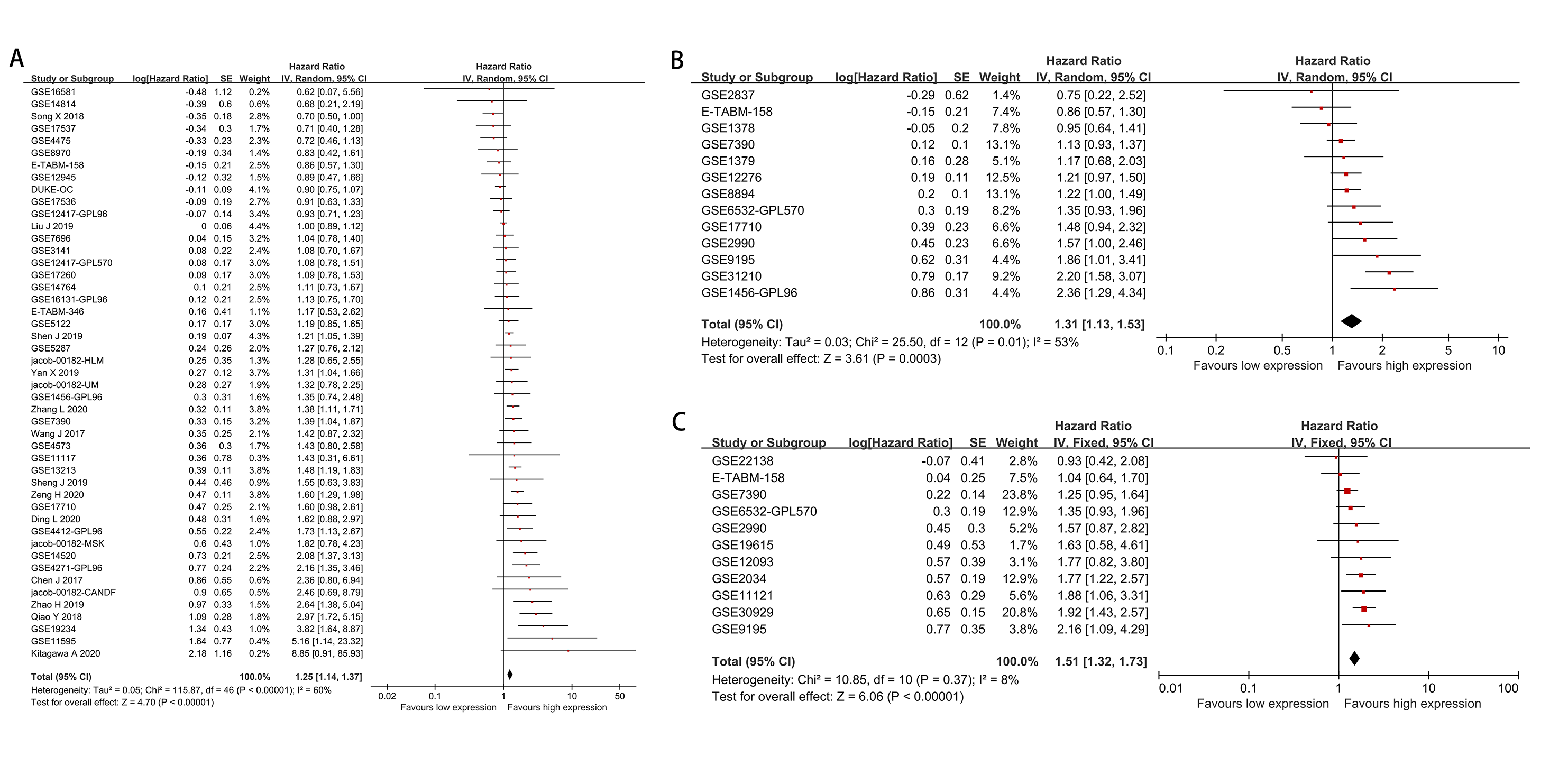

Supplement: Supplementary file 2 [file Image_2.tiff]

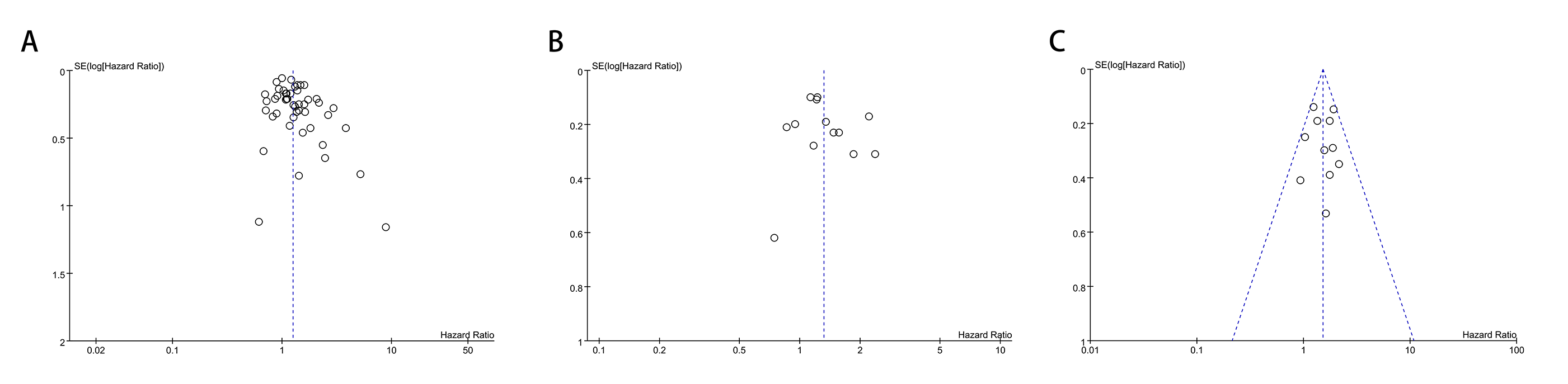

Supplement: Supplementary file 3 [file Image_3.tiff]

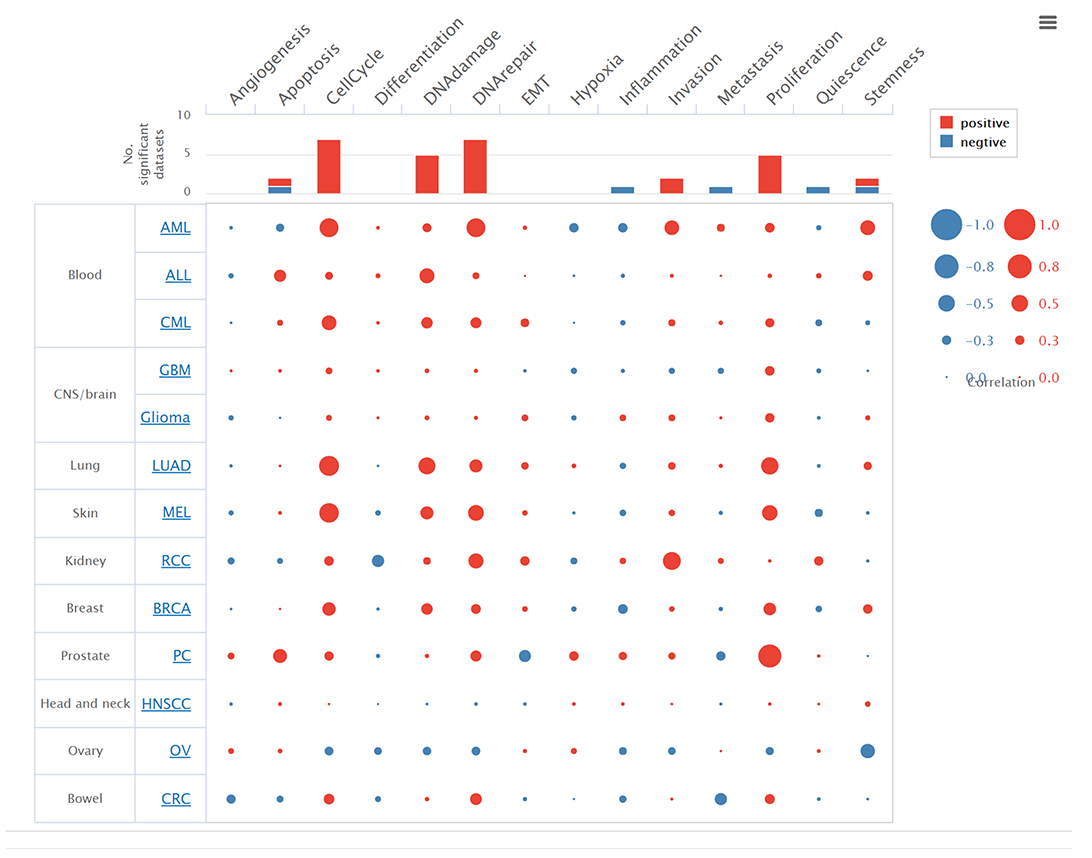

Supplement: Supplementary file 4 [file Image_4.tiff]

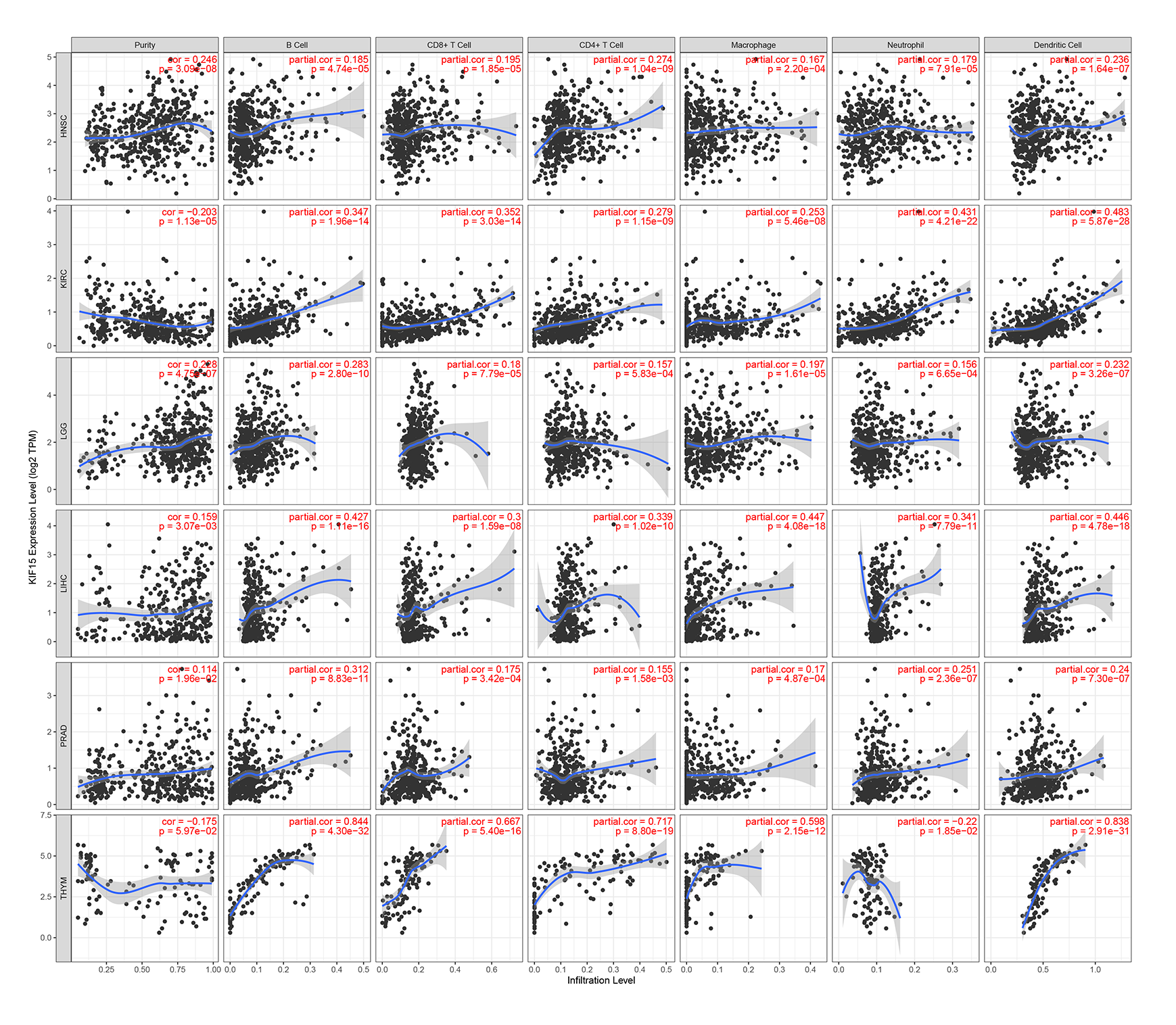

Supplement: Supplementary file 5 [file Image_5.tiff]
