# Supplementary material for: Integrative Pan-Cancer Analysis of KIF15 Reveals Its Diagnosis and Prognosis Value in Nasopharyngeal Carcinoma
Source: Front Oncol. 2022 Mar 11;12:772816. doi: 10.3389/fonc.2022.772816 (PMC8963360; doi:10.3389/fonc.2022.772816)
Supplement: Supplementary file 6 [file DataSheet_1.doc]

## Supplementary Figures

## Supplementary Figure 1. Flow chart of the literature retrieval and screening.

**Supplementary Figure 2.** Forest plot of the survival risk ratio between high and low expression of KIF15 in pan-caner. (A) Overall survival (OS). (B) Relapse-Free Survival (RFS). (C) Distant metastasis-free survival (DMFS).

**Supplementary Figure 3.** Begg’s funnel plots of publication bias of meta-analysis. (A) Overall survival (OS). (B) Relapse-Free Survival (RFS). (C) Distant metastasis-free survival (DMFS).

**Supplementary Figure 4.** The functional state of KIF15 in 13 types of cancer, red plots represent positive correlation, while blue plots represent negative correlation.

**Supplementary Figure 5.** Association of KIF15 mRNA expression with immune cell infiltration levels in different cancer types.

**Supplementary Tables**

Supplementary Table1. The significant difference of KIF15 expression in transcription level between different types cancer and normal tissues.

| **Type** | **N (cancer vs. normal)** | **Fold change** | **T-test** | **p-value** | **References** | **PMID** |
| --- | --- | --- | --- | --- | --- | --- |
| Infiltrating Bladder Urothelial Carcinoma vs. normal | 62 vs. 68 | 2.159 | 7.118 | 1.09E-10 | Lee Bladder | 20233890 |
| Infiltrating Bladder Urothelial Carcinoma vs. normal | 81 vs. 48 | 2.875 | 8.148 | 1.85E-12 | Sanchez-Carbayo Bladder | 16432078 |
| Brain Glioblastoma vs. normal | 542 vs. 10 | 4.534 | 23.663 | 5.07E-14 | TCGA Brain | NA |
| Anaplastic Astrocytoma vs. normal | 19 vs. 23 | 2.400 | 6.988 | 2.16E-8 | Sun Brain | 16616334 |
| Glioblastoma vs. normal | 81 vs. 23 | 2.888 | 10.498 | 8.68E-16 | Sun Brain | 16616334 |
| Oligodendroglioma vs. normal | 50 vs. 23 | 2.024 | 6.555 | 5.92E-9 | Sun Brain | 16616334 |
| Glioblastoma vs. normal | 80 vs. 4 | 5.054 | 12.432 | 3.74E-6 | Murat Brain | 18565887 |
| Anaplastic Oligodendroglioma vs. normal | 23 vs. 6 | 2.351 | 4.408 | 7.46E-5 | French Brain | 16357140 |
| Ductal Breast Carcinoma vs. normal | 40 vs. 7 | 8.979 | 12.984 | 1.19E-14 | Richardson Breast | 16473279 |
| Invasive Breast Carcinoma vs. normal | 76 vs. 61 | 4.732 | 13.608 | 5.46E-27 | TCGA Breast | NA |
| Invasive Ductal Breast Carcinoma vs. normal | 389 vs. 61 | 4.840 | 21.951 | 1.78E-41 | TCGA Breast | NA |
| Invasive Lobular Breast Carcinoma vs. normal | 36 vs. 61 | 3.295 | 9.548 | 4.25E-14 | TCGA Breast | NA |
| Mixed Lobular and Ductal Breast Carcinoma vs. normal | 7 vs. 61 | 3.580 | 7.027 | 6.66E-5 | TCGA Breast | NA |
| Medullary Breast Carcinoma vs. normal | 32 vs. 144 | 2.229 | 11.363 | 1.95E-13 | Curtis Breast | 22522925 |
| Cervical Cancer vs. normal | 20 vs. 8 | 3.840 | 7.601 | 1.39E-9 | Pyeon Multi-cancer | 17510386 |
| Cervical Squamous Cell Carcinoma vs. normal | 32 vs. 24 | 2.438 | 5.149 | 4.04E-6 | Scotto Cervix | 18506748 |
| Rectal Adenocarcinoma vs. normal | 65 vs. 65 | 2.077 | 10.734 | 9.01E-20 | Gaedcke Colorectal | 20725992 |
| Rectal Adenoma vs. normal | 7 vs. 35 | 2.409 | 6.502 | 3.73E-5 | Sabates-Bellver Colon | 18171984 |
| Colon Adenoma vs. normal | 25 vs. 35 | 2.192 | 7.310 | 1.41E-9 | Sabates-Bellver Colon | 18171984 |
| Esophageal Squamous Cell Carcinoma vs. normal | 17 vs. 17 | 2.309 | 6.714 | 6.17E-7 | Hu Esophagus | 20955586 |
| Gastric Intestinal Type Adenocarcinoma vs. normal | 26 vs. 31 | 2.445 | 8.937 | 1.55E-11 | DErrico Gastric | 19081245 |
| Gastric Cancer vs. normal | 80 vs. 80 | 2.089 | 3.968 | 5.51E-5 | Cui Gastric | 20965966 |
| Nasopharyngeal Carcinoma vs. normal | 31 vs. 10 | 2.935 | 7.163 | 2.35E-7 | Sengupta Head-Neck | 16912175 |
| B-Cell Acute Lymphoblastic Leukemia vs. Normal | 10 vs. 5 | -4.470 | -10.867 | 7.99E-6 | Maia Leukemia | 16267031 |
| Chronic Lymphocytic Leukemia vs. Normal | 448 vs. 74 | -2.708 | -21.057 | 1.37E-34 | Haferlach Leukemia | 20406941 |
| Squamous Cell Lung Carcinoma vs. normal | 27 vs. 65 | 4.627 | 16.053 | 2.46E-19 | Hou Lung | 20421987 |
| Lung Adenocarcinoma vs. normal | 45 vs. 65 | 3.287 | 9.712 | 5.87E-14 | Hou Lung | 20421987 |
| Large Cell Lung Carcinoma vs. normal | 19 vs. 65 | 6.809 | 7.728 | 1.33E-7 | Hou Lung | 20421987 |
| Lung Adenocarcinoma vs. normal | 27 vs. 30 | 2.749 | 5.289 | 1.10E-6 | Su Lung | 17540040 |
| Lung Adenocarcinoma vs. normal | 226 vs. 20 | 2.188 | 8.000 | 2.81E-9 | Okayama Lung | 22080568 |
| Unspecified Peripheral T-Cell Lymphoma vs. normal | 28 vs. 20 | 7.495 | 10.937 | 1.25E-14 | Piccaluga Lymphoma | 17304354 |
| Angioimmunoblastic T-Cell Lymphoma vs. normal | 6 vs. 20 | 7.189 | 8.094 | 1.06E-5 | Piccaluga Lymphoma | 17304354 |
| Anaplastic Large Cell Lymphoma vs. normal | 6 vs. 20 | 7.702 | 8.193 | 1.13E-5 | Piccaluga Lymphoma | 17304354 |
| Ovarian Serous Adenocarcinoma vs. normal | 43 vs. 10 | 9.147 | 11.836 | 2.74E-10 | Yoshihara Ovarian | 19486012 |
| Ovarian Serous Cystadenocarcinoma vs. normal | 586 vs. 8 | 3.696 | 16.957 | 2.29E-8 | TCGA Ovarian | NA |
| Myxofibrosarcoma vs. normal | 31 vs. 9 | 2.842 | 12.769 | 3.72E-15 | Barretina Sarcoma | 20601955 |
| Dedifferentiated Liposarcoma vs. normal | 46 vs. 9 | 2.044 | 9.030 | 1.46E-12 | Barretina Sarcoma | 20601955 |
| Pleomorphic Liposarcoma vs. normal | 23 vs. 9 | 3.093 | 9.228 | 1.13E-9 | Barretina Sarcoma | 20601955 |
| Leiomyosarcoma vs. normal | 26 vs. 9 | 2.285 | 7.189 | 4.33E-8 | Barretina Sarcoma | 20601955 |
| Fibrosarcoma vs. normal | 7 vs. 15 | 5.499 | 5.504 | 1.13E-5 | Detwiller Sarcoma | 15994966 |
| Malignant Fibrous Histiocytoma vs. normal | 9 vs. 15 | 3.920 | 4.886 | 4.63E-5 | Detwiller Sarcoma | 15994966 |

Supplementary Table2. The basic characteristics of the studies included in the meta-analysis

| Study/Years | Cancer type | Study type | Outcome  measure | HR extraction |
| --- | --- | --- | --- | --- |
| Chen 2017 | Hepatocellular carcinoma | Retrospective study | OS/RFS | Directly extraction |
| Ding 2020 | Gastric adenocarcinoma | Retrospective study | OS | Indirectly extraction |
| Kitagawa 2020 | Hepatocellular carcinoma | Retrospective study | OS | Indirectly extraction |
| Liu 2019 | Epithelial ovarian carcinoma | Retrospective study | OS/PFS | Directly extraction |
| Qiao 2018 | Lung adenocarcinoma / squamous cell carcinoma | Retrospective study | OS | Indirectly extraction |
| Shen 2019 | Ovarian Carcinoma | Retrospective study | OS | Directly extraction |
| Sheng 2019 | Triple-negative breast cancer | Retrospective study | OS/DFS | Indirectly extraction |
| Song 2018 | Breast cancer | Retrospective study | OS | Directly extraction |
| Wang 2017 | Pancreatic cancer | Retrospective study | OS | Indirectly extraction |
| Yan 2019 | Bladder cancer | Retrospective study | OS/CSS | Directly extraction |
| Zeng 2020 | Breast cancer | Retrospective study | OS/RFS | Directly extraction |
| Zhang 2020 | Lung adenocarcinoma | Retrospective study | OS | Indirectly extraction |
| Zhao 2019 | Bladder cancer | Retrospective study | OS | Directly extraction |

Supplementary Table3. The basic characteristics of the GEO datasets included in the meta-analysis

| GEO dataset | Cancer type | Outcome  measure | HR extraction |
| --- | --- | --- | --- |
| GSE5287 | Bladder cancer | OS | Directly extraction |
| GSE13507 | Bladder cancer | OS | Directly extraction |
| GSE4475 | Blood cancer | OS | Directly extraction |
| GSE8970 | Blood cancer | OS | Directly extraction |
| GSE12417-GPL96 | Blood cancer | OS | Directly extraction |
| GSE12417-GPL570 | Blood cancer | OS | Directly extraction |
| GSE16131-GPL96 | Blood cancer | OS | Directly extraction |
| E-TABM-346 | Blood cancer | OS | Directly extraction |
| GSE5122 | Blood cancer | OS | Directly extraction |
| GSE16581 | Brain cancer | OS | Directly extraction |
| GSE7696 | Brain cancer | OS | Directly extraction |
| GSE4412-GPL96 | Brain cancer | OS | Directly extraction |
| GSE4271-GPL96 | Brain cancer | OS | Directly extraction |
| E-TABM-158 | Breast cancer | OS/RFS/DMFS | Directly extraction |
| GSE1378 | Breast cancer | RFS | Directly extraction |
| GSE2990 | Breast cancer | RFS/DMFS | Directly extraction |
| GSE7390 | Breast cancer | OS/RFS/DMFS | Directly extraction |
| GSE1379 | Breast cancer | RFS | Directly extraction |
| GSE12276 | Breast cancer | RFS | Directly extraction |
| GSE1456-GPL96 | Breast cancer | OS/RFS | Directly extraction |
| GSE6532-GPL570 | Breast cancer | RFS/DMFS | Directly extraction |
| GSE19615 | Breast cancer | DMFS | Directly extraction |
| GSE12093 | Breast cancer | DMFS | Directly extraction |
| GSE2034 | Breast cancer | DMFS | Directly extraction |
| GSE9195 | Breast cancer | RFS/DMFS | Directly extraction |
| GSE11121 | Breast cancer | DMFS | Directly extraction |
| GSE17537 | Colorectal cancer | OS | Directly extraction |
| GSE12945 | Colorectal cancer | OS | Directly extraction |
| GSE17536 | Colorectal cancer | OS | Directly extraction |
| GSE11595 | Esophagus cancer | OS | Directly extraction |
| GSE22138 | Esophagus cancer | DMFS | Directly extraction |
| GSE2837 | Head and neck cancer | RFS | Directly extraction |
| GSE14814 | Lung cancer | OS | Directly extraction |
| GSE3141 | Lung cancer | OS | Directly extraction |
| GSE8894 | Lung cancer | RFS | Directly extraction |
| jacob-00182-HLM | Lung cancer | OS | Directly extraction |
| jacob-00182-UM | Lung cancer | OS | Directly extraction |
| GSE11117 | Lung cancer | OS | Directly extraction |
| GSE4573 | Lung cancer | OS | Directly extraction |
| GSE17710 | Lung cancer | OS/RFS | Directly extraction |
| GSE13213 | Lung cancer | OS | Directly extraction |
| jacob-00182-MSK | Lung cancer | OS | Directly extraction |
| GSE31210 | Lung cancer | OS/RFS | Directly extraction |
| jacob-00182-CANDF | Lung cancer | OS | Directly extraction |
| DUKE-OC | Ovarian cancer | OS | Directly extraction |
| GSE17260 | Ovarian cancer | OS | Directly extraction |
| GSE14764 | Ovarian cancer | OS | Directly extraction |
| GSE26712 | Ovarian cancer | OS | Directly extraction |
| GSE9891 | Ovarian cancer | OS | Directly extraction |
| GSE19234 | Ovarian cancer | OS | Directly extraction |
